# High thoracic anesthesia offers no major benefit over general anesthesia in on-pump cardiac surgery patients: a retrospective study

**DOI:** 10.1186/s40064-016-2541-6

**Published:** 2016-06-21

**Authors:** Michal Porizka, Katerina Koudelkova, Petr Kopecky, Hana Porizkova, Alena Dohnalova, Jan Kunstyr

**Affiliations:** Department of Anaesthesiology, Resuscitation and Intensive Medicine, First Faculty of Medicine, General University Hospital, Charles University in Prague, U Nemocnice 2, 128 08 Prague 2, Czech Republic; Institute of Physiology, First Faculty of Medicine, Charles University in Prague, Albertov 5, Prague 2, 128 00 Czech Republic

**Keywords:** Cardiac surgical procedure, Epidural anesthesia, Patient outcome assessment

## Abstract

**Background:**

Thoracic epidural anesthesia (TEA) has been proposed to improve and facilitate early postoperative outcome in cardiac surgery. The aim of our study was to analyze early postoperative outcome data of patients undergoing cardiac surgery under general anesthesia (GA) with comparison to patients receiving combined TEA and GA.

**Methods:**

Medical records data from 288 patients who underwent elective on-pump cardiac surgery were retrieved and analyzed. Patients were divided into two study groups according to the type of anesthesia used: GA group (n = 141) and TEA group (n = 147). Early postoperative outcome data including quality of analgesia and major organ outcome parameters were compared between the study groups.

**Results:**

There was no major difference in early postoperative outcome data between the study groups, except for shorter time to extubation (6.0 ± 10.0 vs. 6.9 ± 8.8 h, respectively, P < 0.05) and hospital stay (10.7 ± 5.9 vs. 12.9 ± 8.8 days, respectively, P < 0.05) in TEA group compared to GA group. Also TEA group as compared to GA group had lower pain numeric rating scale scores (1 ± 1.1 vs. 1.4 ± 1.5 at 24 h, respectively, P < 0.05) and morphine requirements during the first 24 h after surgery (148.2 vs. 193 ± 85.4 μg/kg, respectively, P < 0.05).

**Conclusion:**

Both anesthetic methods were equivalent in most postoperative outcome measures. Thoracic epidural analgesia provided superior pain relief, shorter time to extubation and earlier hospital discharge.

## Background

Epidural anesthesia is effectively applied in different types of surgery to improve perioperative outcome (Block et al. 2003). In cardiac surgery, high thoracic epidural anesthesia (TEA) offers several advantages including thoracic sympathicolysis, attenuated stress response and myocardial blood flow redistribution (Chaney 2006). In previous studies, these effects were coupled with improved analgesia (Liu et al. 2004), earlier extubation time (Liu et al. 2004), less respiratory complications (Liu et al. 2004; Ballantyne et al. 1998), decreased incidence of arrhythmias (Svircevic et al. 2011) and postoperative myocardial infarction (Beattie et al. 2001). However, as recent meta-analyses show, no mortality benefit has been proven so far (Svircevic et al. 2013).

Despite these advantages, the application of TEA in clinical practice is more or less limited because of its potential risk of adverse events such as epidural hematoma or abscess resulting in spinal cord compression (Hemmerling et al. 2013). Furthermore, TEA may also be a risk factor for arterial hypotension and hemodynamic instability in the postoperative period (Gramigni et al. 2013).

In this retrospective study, we aimed to compare the effects of conventional general anesthesia (GA) and combined TEA and GA on early postoperative outcome measures including the quality of analgesia, hemodynamic stability and perioperative mortality in patients undergoing elective cardiac surgery with cardio-pulmonary bypass.

## Methods

This study was approved by local ethics committee of General University Hospital in Prague.

### Patient population

All adult patients undergoing any elective cardiac surgical procedure with the use of cardio-pulmonary bypass from January 2012 through September 2012.

### Data retrieval

Data from hospital records and local registry were collected and reviewed retrospectively. Patient demographics, preoperative status, operative risk assessment calculated by EuroSCORE II scoring system (ES II), operative and early postoperative outcome data were retrieved and analyzed. The latter included major organ system outcome parameters, length of hospital stay, perioperative mortality and quality of analgesia, which was evaluated using Numeric rating scale (NRS) scoring at rest recorded every 6 h for 3 days postoperatively. The procedure was considered to be elective if the patient was admitted to hospital 1 day prior to surgery excluding all the acute conditions as defined by ES II criteria.

### Study protocol

All patients included in the study were divided into two groups according to the type of anesthesia used. The first group (GA group) comprised of patients undergoing cardiac surgery in sole general anesthesia (GA), receiving conventional, protocol-based opioid analgesia postoperatively. The second group (TEA group) consisted of patients undergoing surgery under combined TEA and GA and receiving postoperative epidural analgesia. All retrieved patient’s data were then analyzed and compared between the two study groups.

### Premedication and anesthetic technique

There is a standard premedication and anesthetic protocol for cardiac surgical patients in our institution. All patients receive 0.25–0.5 mg of alprazolam orally 1 h prior to arrival to the operating room. General anesthesia is induced with an intravenous bolus of propofol (1.5–2 mg/kg), sufentanil (0.5 μg/kg) and rocuronium (0.4–0.6 mg/kg). GA is maintained using isoflurane of minimal alveolar concentration 0.7–1.0 in a gas mixture of oxygen and air. Additional boluses of 25–50 μg of sufentanil are administered in case of analgesic insufficiency. No other myorelaxation is used throughout the procedure.

The epidural puncture was performed in the TEA group at the level Th1/2–Th2/3 using an 18-gauge Tuohy epidural needle (Perican, B. Braun, Melsungen, Germany) under local anesthesia in sitting or lateral decubitus position before induction of anesthesia. Coagulation profiles of all patients were normal before epidural puncture. The epidural space was identified using hanging drop or loss of resistance technique and 10 mL of 0.25 % bupivacaine was administered as a bolus into the space. Afterwards, an epidural catheter (Perifix-Katheter, B. Braun, Melsungen, Germany) was inserted 2–4 cm into the epidural space. The level of anesthesia was determined by loss of pinprick sensation (Th1–Th10). Then, continuous epidural infusion using plain 0.25 % bupivacaine was applied with a rate of 7–10 mL/h till the end of surgery. After epidural puncture GA was induced and maintained using isoflurane in the same dosage as in the GA group. When required additional sufentanil was administered according to the pain response.

### Postoperative management

After the transfer to ICU, all patients were weaned off the ventilator and extubated according to local extubation protocol. This included fully awake, cooperative patients with stable hemodynamic parameters, without significant blood loss (<200 mL/2 h) and with acceptable arterial blood gases parameters (i.e. PaO_2_ > 60 mmHg and PaCO_2_ < 60 mmHg) on non-aggressive ventilation (pressure support ventilation, PEEP ≤ 5 cm H20, FiO_2_ ≤ 0.4, pressure support ≤6 cm H_2_0 and respiration frequency ≥10/min). On the first postoperative day, pain management in the GA group was conducted by intravenous administration of 1 g of paracetamol every 6 h combined with a nurse-driven intravenous morphine protocol. Patients with NRS scores <5 received 1 mg of intravenous morphine and 2 mg of morphine were administered to patients with NRS scores >5. The minimal time interval between 2 morphine injections was 10 min and the maximal dose was 20 mg per 12 h. In case of inadequate analgesia further on, additional morphine was administered on doctor’s request.

After 24 h analgesic therapy continued with 1 g of oral paracetamol six hourly and 20 mg of oral oxycodone twelve hourly. Postoperative analgesia in the TEA group was provided by continuous infusion of local anesthetics to epidural catheter (0.1 % bupivacaine by rate of 3–8 mL/h) supplemented by the same paracetamol regimen as in the GA group. In case of epidural analgesia insufficiency, opioids were administered by the identical protocol as in the GA group.

Antiplatelet/anticoagulation therapy was started 6 h after the surgery if blood loss was less than 20 mL/h. Epidural catheter was removed on the fourth postoperative day with respect to safe withdrawal intervals of anticoagulants, i.e. 4 h after stopping continuous infusion of unfractionated heparin, 12 h after last prophylactic dose of low-molecular weight heparin and 24 h after last therapeutic dose of low-molecular weight heparin. Chest tubes were removed on the second or third postoperative day.

Criteria for intensive care unit discharge were as follows: fully alert and cooperative patient without significant neurological impairment, hemodynamic stability without inotropic or vasopressor therapy, no hemodynamically significant arrhythmias, spontaneous breathing with arterial oxygen saturation >90 % at FiO2 ≤ 50 % via a facemask, urine output >0.5 mL/kg/h, chest tube drainage <20 mL/h. Criteria for hospital discharge were as follows: hemodynamically stable with controlled arrhythmias, independent in ambulation and feeding, afebrile with no infections and clean wound, normal voiding and bowel movements, full oral diet, pain controlled by oral medication.

### Statistical analysis

Data are presented as mean ± standard deviation. SPSS 13.0 software (SPSS Inc., Chicago, IL, USA) was used for statistical analysis. A Chi square test was used for comparisons of preoperative and postoperative qualitative parameters, followed by Fisher’s exact test. Normal distribution was tested for all quantitative parameters. Mann–Whitney non-parametric test was used for comparisons of quantitative parameters between the study groups. P values <0.05 were considered statistically significant.

## Results

There were 288 patients included in the study, 141 in the GA group and 147 in the TEA group.

### Demographic, preoperative and peroperative data

 There was no difference in demographic and preoperative data between the study groups, except for higher proportion of older patients and higher incidence of left ventricular systolic dysfunction in the GA group as compared to the TEA group (Table 1). Also no difference was found in the type of antihypertensive medication administered prior to surgery in the TEA group compared to the GA group: B-blockers (66.7 and 67.4 % respectively, P = 0.99), angiotensin-converting enzyme inhibitors (44.2 and 49.6 %, P = 0.65), sartans (15.6 and 12.1 % respectively, P = 0.68) and calcium-channel blockers (23.8 and 19.9 % respectively, P = 0.72).

**Table 1:** Demographics and preoperative data

	GA, n = 141	TEA, n = 147	P value
EUROSCORE II	3.5 ± 3.8	3.65 ± 3.64	0.694
Age (years)	66.9 ± 10.9	64.3 ± 9.8	*0.041*
Height (meters)	1.70 ± 0.09	1.71 ± 0.09	0.137
Weight (kilograms)	80.5 ± 16.3	82.9 ± 17	0.104
BSA (m^2^)	1.91 ± 0.20	1.96 ± 0.21	0.064
BMI (kg/m^2^)	27.8 ± 4.5	28.4 ± 5.5	0.395
Male (female)	94 (47)	99 (48)	0.902
CAD	82 (58.2 %)	76 (51.7 %)	0.066
Arterial hypertension	114 (80.9 %)	109 (74.1 %)	0.208
LV EF (%)	54.4 ± 12.6	56.8 ± 11.2	*0.044*
RV dysfunction	14 (9.9 %)	15 (10.2 %)	0.996
COPD	32 (22.7 %)	40 (27.2 %)	0.295
Diabetes mellitus	27 (19.1 %)	38 (25.9 %)	0.832
Stroke/TIA	15 (10.6 %)	16 (10.9 %)	0.946
NYHA			0.370
I	10 (7.1 %)	14 (9.5 %)	
II	76 (53.9 %)	83 (56.5 %)	
III	52 (36.9 %)	46 (31.3 %)	
IV	3 (2.1 %)	4 (2.7 %)	
Serum creatinine (µmol/L)	91.6 ± 27.4	94.4 ± 35.7	0.999

*EUROSCORE II* European System for Cardiac Operative Risk Evaluation II, *BSA* body surface area, *BMI* body mass index, *CAD* coronary artery disease, *LV EF* left ventricular ejection fraction, *RV* right ventricular, *COPD* chronic obstructive pulmonary disease (FEV1 ˂80 %, FEV1/FVC˂70 %), *TIA* transitory ischemic attack, *NYHA* New York Heart Association heart failure classification

Italic values indicate significance value of P < 0.05

Operative data analysis revealed higher incidence of aortic valve replacement, aortic surgery and reoperations in the TEA group (Table 2). On the contrary, more coronary artery bypass grafting procedures were performed in the GA group (Table 2). No other significant differences in operative data including aortic cross-clamp time and length of cardio-pulmonary bypass were noted. Operative risk severity, as assessed by EUROScore II, was similar in the two study groups, without a significant difference (Table 2).

**Table 2:** Operative data

	GA, n = 141	TEA, n = 147	P value
Type of surgery			
CABG	44 (31.2 %)	23 (15.6 %)	*0.002*
AVR	22 (15.6 %)	37 (25.2 %)	*0.044*
MVR	8 (5.7 %)	4 (2.7 %)	0.210
MV repair	4 (2.8 %)	9 (6.1 %)	0.783
Aortic surgery	4 (2.8 %)	13 (8.8 %)	*0.031*
Combined procedure	50 (35.5 %)	54 (36.7 %)	0.561
Other	9 (6.4 %)	7 (4.9 %)	0.210
Reoperation	3 (2.1 %)	12 (8.2 %)	*0.021*
Re-exploration for			
Bleeding	3 (2.1 %)	2 (1.4 %)	0.618
Tamponade	0	2 (1.4 %)	0.586
Blood loss (mL)	915 ± 340	964 ± 457	0.311
Aortic cross clamp time (min)	75 ± 33	78 ± 41	0.847
CPB time (min)	111 ± 41	118 ± 62	0.961

*CABG* coronary artery bypass grafting, *AVR* aortic valve replacement, *MVR* mitral valve replacement, *MV* mitral valve, *TV* tricuspid valve, *CPB* cardio-pulmonary bypass

Italic values indicate significance value of P < 0.05

The total dose of sufentanil administered during surgery was significantly lower in the TEA group compared to the GA group (0.65 ± 2.21 and 2.67 ± 0.83 µg/kg respectively, P < 0.05).

### Quality of analgesia

**Fig. 1:**
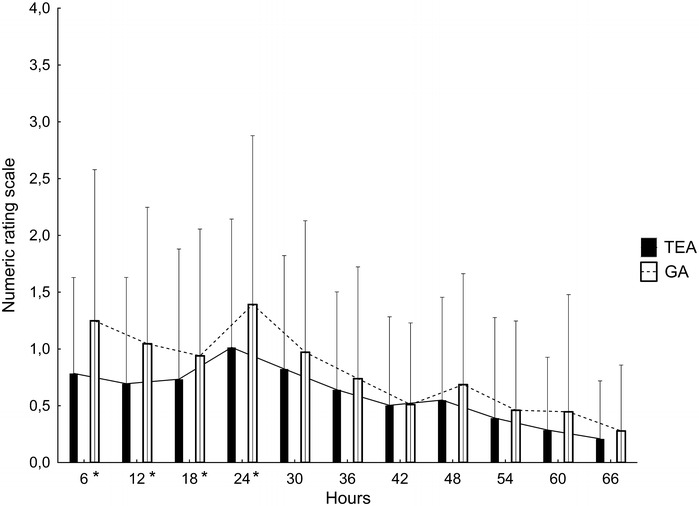
Mean postoperative pain scores at rest by group (TEA, *black*; GA, *white*) and time (hours after surgery). Data are presented as mean ± standard deviation. *TEA* thoracic epidural anesthesia, *GA* general anesthesia, *NRS* numeric rating scale. *P < 0.05

NRS scores were significantly lower at 6, 12, 18, 24 h after surgery in the TEA group compared to the GA group. Subsequently, in the following 48 h, NRS scores did not differ between the study groups (Fig. 1). The total morphine requirements were lower in the TEA group compared to the GA group (148.2 ± 82.5 and 193 ± 85.4 µg/kg respectively, P < 0.05).

### Postoperative outcome data

There was no difference in all major organ outcome parameters between the study groups (Tables 3, 4). Total dose of norepinephrine and duration of vasopressor support tended to be lower in the TEA group compared to the GA group, but did not reach a statistical significance (Table 3). Time to extubation was significantly lower in the TEA group compared to the GA group (Table 3).

**Table 3:** Cardiovascular and respiratory complications

	GA, n = 141	TEA, n = 147	P value
Myocardial infarction	1 (0.7 %)	0	0.490
Low cardiac output	5 (3.5 %)	9 (6.1 %)	0.309
Ionotropes	28 (19.9 %)	33 (22.4 %)	0.591
IABP	3 (2.1 %)	7 (4.8 %)	0.336
Atrial fibrillation	37 (26.2 %)	44 (29.9 %)	0.486
NE support	92 (65.2 %)	100 (68 %)	0.882
NE > 0.1 µg/kg/min	29 (20.6 %)	31 (21.1 %)	0.806
NE total dose (µg/kg)	211 ± 807	123 ± 289	0.260
NE support duration (h)	28 ± 36	21 ± 29	0.114
ICU fluid balance (mL)	3890 ± 1936	3518 ± 2405	0.124
Lung atelectasis	6 (4.3 %)	8 (5.4 %)	0.640
Pneumonia	1 (0.7 %)	4 (2.7 %)	0.371
Time to extubation (h)	6.9 ± 8.8	6.0 ± 10.0	*0.002*
Reintubation	4 (2.8 %)	2 (1.4 %)	0.428

*IABP* intra-aortic balloon pump, *NE* norepinephrine, *TIA* transitory ischemic attack, *ICU* intensive care unit

Italic value indicates significance value of P < 0.05

**Table 4:** Renal, gastrointestinal, neurological and infectious complications

	GA, n = 141	TEA, n = 147	P value
Acute renal failure	5 (3.5 %)	4 (2.7 %)	0.746
CRRT	2 (1.4 %)	2 (1.4 %)	1.000
Peak serum creatinine (µmol/L)	99.6 ± 37.1	102.4 ± 40.3	0.864
Upper gastrointestinal bleeding	1 (0.7 %)	1 (0.7 %)	1.000
Mesenterial ischemia	1 (0.7 %)	1 (0.7 %)	1.000
Stroke/TIA	3 (2.1 %)	2 (1.4 %)	0.679
ICU delirium	8 (5.7 %)	7 (4.8 %)	0.728
Blood stream infection	0	2 (1.4 %)	0.498
Urinary tract infection	0	0	
Surgical site infection	3 (2.1 %)	0	0.116

*CRRT* continuous renal replacement therapy, *TIA* transitory ischemic attack, *ICU* intensive care unit

### Length of hospital stay and early mortality

There was a shorter hospital stay in the TEA group compared to the GA group, however no difference was found in the ICU length of stay between the study groups (Table 5). Also no significant difference in ICU or hospital mortality was noted (Table 5).

**Table 5:** Mortality and length of ICU/hospital stay

	GA, n = 141	TEA, n = 147	P value
ICU mortality	3 (2.1 %)	4 (2.7 %)	1.000
Hospital mortality	5 (3.5 %)	5 (3.4 %)	0.998
Length of ICU stay (days)	5.5 ± 4	5.3 ± 3.1	0.814
Length of hospital stay (days)	12.9 ± 8.8	10.7 ± 5.9	*0.019*

*ICU* intensive care unit

Italic value indicates significance value of P < 0.05

No serious complications of epidural catheter insertion, including clinically significant epidural hematoma or abscess were identified.

## Discussion

Our retrospective analysis showed that the use of high TEA was associated with shorter time to extubation, reduced length of hospital stay and superior analgesia in comparison to GA in patients undergoing elective on-pump cardiac surgery. Other major organ outcome parameters including early mortality did not differ between the study groups.

Since its first use in cardiac surgery in Clowes et al. (1954), TEA has been used primarily to provide reliable postoperative analgesia. Pain management in postoperative period is one of the most essential components of postsurgical patients care and insufficient analgesia may lead to many unfavorable outcome, including hemodynamic instability, impaired immune response, extensive catabolism, and hemostatic disorders (Weissman 1990). Epidural anesthesia in cardiac surgery provides superior pain relief in comparison to standard intravenous opioid treatment (Liu et al. 2004) and our study results confirm these findings. However, we found that analgesic efficacy of TEA was better only in the immediate postoperative period during the first 24 h (Fig. 1). Afterwards pain scores did not differ between the study groups which is also in agreement with previous reports (Clowes et al. 1954). Concomitantly, patient's morphine requirements were significantly lower in the TEA group. Deleterious effects of opioid analgesia include respiratory depression, sedation, vomiting and nausea, constipation, urinary retention, pruritus and ileus and may finally worsen patient's postoperative outcome (Mehta and Arora 2014). Therefore, from this point of view, the use of TEA seems to be also advantageous.

In accordance with earlier studies (Liu et al. 2004), we found that the time to extubation was significantly shorter in patients with TEA (Table 3). Early extubation in conjunction with effective analgesia and rapid mobilization constitutes a cornerstone of fast-tracking concept in anesthesia (Nanavati and Prabhakar 2014) and forms a necessary basis especially for prevention of postoperative respiratory complications. Postoperative pulmonary dysfunction (PPD) is common and significant after cardiac surgery, as it contributes to increased morbidity, mortality and prolongs hospitalization stay (Wynne and Botti 2004). Its clinical manifestations include pleural effusion, atelectasis, postoperative hypoxemia and acute respiratory distress syndrome. Pathophysiology of PPD is complex and its mechanisms are not fully understood. However, the most significant factors include surgery related factors, effect of general anesthesia with mechanical ventilation, cardiopulmonary bypass and systemic inflammatory response syndrome (Badenes et al. 2015). Additionally, postoperative pain and impairment of diaphragmatic function are also important determinants of such pulmonary dysfunction (Diehl et al. 1994). Nevertheless, in our study, better analgesia and shorter ventilation times in the TEA group did not result in reduced incidence of these respiratory complications, as they were equally frequent in both study groups (Table 3). That is in contrast to the latest meta-analysis that showed a lower risk of respiratory events for patients receiving TEA during surgery compared with those receiving GA alone (Svircevic et al. 2013). However, in a recent study, it was demonstrated that an early extubation (within 9 h after cardiac surgery) is associated with an improved outcome and was shown to be the best predictor of uncomplicated recovery (Camp et al. 2009). Even though our data show that time to extubation was shorter in the TEA group, this reduction was only of a mild degree (6.0 h in TEA group vs. 6.9 h in GA group). Thus, both study groups generally fulfilled early extubation criteria, as identified by the above mentioned study, possibly having a favorable impact on postoperative outcome in both groups. Similarly, pain relief was significantly better in the TEA group during the first postoperative day, however pain scores in both groups were generally low (average NRS at 24 h of 1.4 ± 1.5 in GA group vs. 1 ± 1.1 in TEA group, P < 0.05), representing also a mild degree of postoperative pain. These results show that morphine analgesia in the GA group provided sufficient pain relief and also enabled early extubation, resulting in similar incidence of PPD as in the TEA group.

Furthermore, the rest of major postoperative outcome parameters including neurologic complications, renal impairment, myocardial dysfunction, infective complications and perioperative mortality did not differ between the study groups either, which corresponds to the results of a recent, large meta-analysis (Svircevic et al. 2013). On the other hand, we also did not notice any reduction in the incidence of supraventricular arrhythmias which has been reported by our previous study with TEA awake patients (Porizka et al. 2011) and a meta-analysis (Svircevic et al. 2013). However, when looking at these published data from meta-analysis more closely, we discover that in the large study by Scott et al. (2001) B-blockers were discontinued 5 days preoperatively, which must have contributed significantly to increased incidence of postoperative arrhythmias. Subsequently, studies published since then have been unable to repeat these results (Hansdottir et al. 2006). All patients in our study were on the regular, anti-hypertensive medication including B-blockers until the day of surgery and their postoperative administration was restored as soon as possible. This may be the reason, why the incidence was similar in both study groups, as many recent studies have shown that postoperative supraventricular tachyarrhythmias can be reduced just with the use of B-blockers, amiodarone or atrial pacing (Burgess et al. 2006).

Many factors other than type of anesthesia, including comorbidities, age, acuity and type of operative procedure, influence final perioperative outcome. In our study, there were a few significant differences in preoperative and operative data (Tables 1, 2) in the study groups. Nevertheless, most of these parameters are included in final operative risk severity score calculation, in our case widely used ES II which was validated in large populations of cardiac surgical patients (Biancari et al. 2012). ESII was similar in both study groups (3.5 % in GA group and 3.65 % in TEA group), therefore we assume that these study groups were comparable. On the other hand, such ESII scores represent relatively low risk category of cardiac surgical patients. This may be the reason why we did not observe beneficial effects of epidural anesthesia (lower incidence of pulmonary complications and supraventricular arrhythmias) reported by recent meta-analyses (Svircevic et al. 2011, 2013) and these effects might become more apparent when higher risk patients, especially those with pulmonary or cardiac dysfunction, are involved.

As mentioned above, TEA represents one of the possible methods of fast-track anesthesia and there are studies reporting shorter length of hospital stay when using TEA (de Vries et al. 2002). From that point of view, we present conflicting results. In our study, there was no difference in the duration of ICU stay, while hospital stay was shorter in the TEA group (Table 5). Considering that preoperative characteristics, most of operative data and early postoperative outcome did not differ between the study groups, it is most likely that this observation was not caused by TEA alone, as no epidural analgesia was used during the ward stay beyond the fourth postoperative day. On the other hand, other factors that are not related to patient’s characteristics or anesthetic/surgical method used may play a role. These represent the local protocol of patient hospital discharge, which includes a factor of surgeon’s own decision process and preferences. Also a social aspect, reflecting whether the patient is discharged from hospital to home/other medical facility and availability of spa care, is of significant importance and may finally affect the total length of hospital stay.

Despite all these potentially beneficial effects of TEA, its application in connection with cardiac surgery still remains very controversial. The widespread safety concern of TEA is whether it promotes an extensive sympathectomy with the resultant hypotension, hemodynamic instability and increased use of vasopressors. However, as both experimental (Taniguchi et al. 1997) and human (Magnusdottir et al. 1999) data show, sympathetic block does not extend below the sensory block, which typically extends from T1 to T8, and sympathetic tone is preserved in large splanchnic and limb vascular beds. In our study, hemodynamic stability and degree of hypotension were evaluated by the retrospective assessment of norepiephrine (NE) use and the net fluid balance. No significant difference was found in the number of patients requiring NE support and the incidence of high level of NE support (>0.1 µg/kg/min) between the study groups (Table 3). There was a strong trend towards shorter duration and lower total dose of NE support in the TEA group, but it was not statistically significant (Table 3). We also evaluated the total net fluid balance at the end of ICU stay, as fluid therapy constitutes the first line treatment in hypotensive states and therefore may reflect the degree of postoperative hemodynamic instability. Our results also show no major statistical difference in the net fluid balance between the study groups (Table 3). Nevertheless, there are other major factors associated with hypotension in the immediate postoperative period that may affect the final fluid balance and the use of vasopressors. These include the degree of blood loss, myocardial dysfunction with the need for inotropes, incidence of sepsis and length of cardio-pulmonary bypass (Fischer and Levin 2010). All these parameters did not differ between the study groups (Tables 2, 4) either. Additionally, antihypertensive medication administered on the day of surgery may also affect hemodynamic stability and need for vasopressors in perioperative period (Auron et al. 2011), however there were no differences in the type of antihypertenzives given preoperatively in the study groups. Thus, all these findings indicate that the degree of hypotension is not increased and hemodynamic stability is preserved in patients with TEA after cardiac surgery.

Another major concern associated with the use of TEA in cardiac surgery is the risk of epidural hematoma or abscess formation with possible catastrophic neurological sequelae and there is clearly a big reluctance among anesthesiologists to place epidural catheters in these patients. Nevertheless, the latest epidural hematoma risk assessment in cardiac surgery stated a risk of 1:5493 (Hemmerling et al. 2013) which is comparable to the risk in non-obstetrical surgery (Volk et al. 2012), and is generally considered to be relatively low. In the present study, we did not experience any of the above described serious complications in our TEA patients. On the other hand, all possible precautions must be undertaken to minimize this risk, including the acceptance of only normal coagulation parameters and adequate withdrawal intervals of antithrombotic drugs before epidural puncture and insertion of epidural catheter (Gogarten et al. 2010). The time interval between epidural catheter placement and full heparinization in on-pump cardiac surgery should be minimally 1 h (Mehta and Arora 2014; Gogarten et al. 2010).

Our study has a few limitations. The major one is its retrospective design and relatively small number of patients, especially for an analysis of mortality data. When evaluating the hemodynamic stability between the study groups, we used only indirect markers including use of NE and the net fluid balance, as the measurements of cardiac output and other derived hemodynamic measurements including systemic vascular resistance were not routinely performed and calculated in every patient.

In conclusion, in this retrospective observational study, the effectiveness of TEA combined with general anesthesia followed by continuous epidural analgesia and the effectiveness of general anesthesia alone followed by the nurse-driven intravenous morphine analgesia were compared with respect to quality of analgesia and postoperative complications after cardiac surgery. Except for superior analgesia, TEA did not offer any major advantage concerning quality of recovery and morbidity compared to GA.
